# Surgical and Practical Observations on the Diseases of the Human Foot, with Instructions for Their Treatment. To Which Is Added Advice on the Management of the Hand

**Published:** 1845-10

**Authors:** 


Art. IV.—*Surgical and Practical Observations on the Diseases of the Human Foot, with Instructions for their Treatment. To which is added Advice on the Management of the Hand.* By John Eisenberg.—*London*, 1845. 4to, pp. 252.

This is the most splendidly got-up book that has come under our notice since we commenced our career. It is a real luxury to sexagenarian eyes to look on its silk-soft, silk-white paper, its beautiful black, tall type, its gorgeous promenades of margin, twice the extent of the parterre of text. But, alas, it is all fairy ware—fine semblance, no substance; and we warn our readers all and sundry against meddling with the charming cheat. It is a puff in quarto, an advertisement of 250 pages, all to the honour and glory of John Eisenberg. It surely missed its way when it fell into ours. It may satisfy or mystify the learned lords and ladies who read Dr. Cronin and Dr. Riadore—but it has nothing in common with the principles or objects of 'The British and Foreign Medical Review.' Does the author speak the truth in stating in his dedication to Dr. Marshall Hall, that he, Dr. Hall, had "accepted" the dedication, and "considered" the author's "labours" as "not unworthy of his kind protection?" We hope for the sake of Dr. Hall, that this, like much of the book, is a fib.

